# What to do when the second twin is non‐vertex?

**DOI:** 10.1111/aogs.15067

**Published:** 2025-02-07

**Authors:** Katrine Vasehus Schou, Marianne Johansen

**Affiliations:** ^1^ Department of Obstetrics and Gynecology, Copenhagen University Hospital Rigshospitalet Copenhagen Denmark

The incidence of twin deliveries is still increasing in many countries, as a consequence of higher maternal age, and assisted reproduction technology. The choice of planned mode of delivery in twin gestations and intrapartum clinical management are still debated with many discrepancies between countries and centers.

A lot of factors influence the physician's recommendation for mode of delivery in a twin gestation. In high income countries there seems to be consensus that vaginal delivery is a reasonable and likely the preferred option for delivery in near term uncomplicated twin pregnancies with the first twin in a vertex presentation.^1^, ^2^ For many years it has been assumed that trial of labor (TOL) with the second twin being in a non‐vertex presentation is associated with a higher maternal and neonatal risk, including an increased risk of combined delivery, and hence maternal and neonatal morbidity. However, more recent research has identified other independent risk factors such as higher gestational age, nulliparity, and the use of medical induction of labor. Several studies have now confirmed that TOL in twin pregnancies is a safe option, irrespective of second twin presentation if certain well‐defined criteria are met, including a recent ultrasound regarding fetal position and estimated fetal weight focusing on weight con‐ or dis‐cordance, and with the appropriate infrastructure and clinical expertise being accessible on labor ward. A sub‐analysis of The Twin Birth Study, however, found that transverse/oblique lie of twin B after the birth of twin A was a risk factor for combined delivery and combined delivery a risk factor for adverse neonatal outcome.^1^ In this context it is important to remember that the majority of second twins present in a vertex position and that up to one tenth of second twins in non‐vertex presentation experience a spontaneous version during labor affecting the likelihood of successful vaginal delivery.^3^ Still, up to 4% of women opting for vaginal twin delivery ends up with “the worst of two worlds” going through a combined delivery with the burden of undergoing a vaginal delivery followed by an emergency cesarean section with its inborn risks. When choosing the most appropriate mode of delivery, this potential scenario of combined delivery seems to affect women's choice.

Almost 9 out of 10 second twins starting labor in a non‐vertex presentation (approximately one third of all twins) end up in a final non‐vertex presentation and may challenge the attending obstetrician with a decision on how to deliver. In some centers the clinicians choose to perform an intrapartum external version (external cephalic version [ECV]) or correction of fetal position of the non‐vertex second twin, to achieve a vertex presentation, whilst others opt for either spontaneous breech delivery or breech extraction, whichever comes most natural, in order to achieve a safe vaginal delivery. Some centers, again, may opt for cesarean delivery of the second twin when in a non‐vertex position. Furthermore, internal podalic version of the second twin before breech extraction is used as an option with vertex, oblique, or transverse presentation of the second twin; especially when urgent delivery is required. The lack of experience in breech deliveries, in general, may influence the clinician's choice of mode of delivery of the second twin in a final non‐vertex presentation. There is also some resistance or fear to perform intrauterine maneuvers amongst obstetricians, which may also affect the decision on delivery mode. Another factor that may influence the choice is chorionicity as monochorionic twins need shorter intertwin birth interval due to the small risk of intrapartum transfusion syndrome.

In this edition of *AOGS*, Dymon et al.^4^ presents a systematic review on intrapartum ECV of the second twin when presenting in a final non‐vertex presentation by analyzing success rate and adverse outcomes in terms of neonatal death, major birth trauma, or Apgar score <7 at 5 min. Ten studies of older date (1983–1998) are included in the study and the overall success rate for ECVs is 64.4% (*n* = 289 attempts) with success rates varying between 42% and 80%. Nine out of ten of successful ECVs ended up in vaginal birth. Approximately half of the unsuccessful ECVs gave vaginal birth to the second twin in a breech presentation, whereas the other half of the failed attempts of ECV were delivered by cesarean section. This systematic review has many limitations, especially due to its retrospective and historic nature, as well as scarce, low‐quality data from the available studies included. Based on this systematic review it is therefore difficult to draw meaningful and evidence‐based conclusions on efficacy and safety of the use of intrapartum ECV as a clinical management maneuver for the non‐vertex second twin. Furthermore, the study does not address the outcome of the spontaneous breech deliveries of the second twin in order to discuss the pros and cons for trial of ECV, nor is the indication for cesarean section in failed ECVs discussed, and it remains unclear whether vertex presentation of the second twin would have made a difference. However, the study findings do suggest that intrapartum ECV of the second twin with a final non‐vertex presentation may have a role to play as an integral part of the toolbox for clinical management of the final non‐vertex presenting second twin. More studies, preferably prospective and ideally of randomized controlled trial (RCT)‐design, are needed to qualify the full picture of optimal management of TOL for twin deliveries with the second twin being in a final non‐vertex presentation. RCTs are, however, not easy to conduct in complex clinical scenarios like a twin delivery. For now, we, as obstetricians, must remain clinically updated, and trained in intra‐ and extrauterine maneuvers to be prepared for every possible clinical scenario of a vaginal twin delivery with a toolbox consisting of all the above‐mentioned maneuvers including the ECV of a non‐vertex presenting second twin.

Simulation based clinical skills training of vaginal twin delivery for healthcare professionals at the labor wards, could be beneficial to prepare for all possible scenarios that may arise in real‐life. This method may effectively complement clinical experience, helping clinicians develop, and maintain the expertise necessary to safely manage twin vaginal deliveries.
